# The multifaceted roles of embryonic microglia in the developing brain

**DOI:** 10.3389/fncel.2023.988952

**Published:** 2023-05-12

**Authors:** Yuki Hattori

**Affiliations:** Department of Anatomy and Cell Biology, Graduate School of Medicine, Nagoya University, Nagoya, Japan

**Keywords:** microglia, brain, neurogenesis, neuron, macrophage, blood vessel

## Abstract

Microglia are the resident immune cells of the central nervous system (CNS). Microglia originate from erythromyeloid progenitors in the yolk sac at the early embryonic stage, and these progenitors then colonize the CNS through extensive migration and proliferation during development. Microglia account for 10% of all cells in the adult brain, whereas the proportion of these cells in the embryonic brain is only 0.5–1.0%. Nevertheless, microglia in the developing brain widely move their cell body within the structure by extending filopodia; thus, they can interact with surrounding cells, such as neural lineage cells and vascular-structure-composing cells. This active microglial motility suggests that embryonic microglia play a pivotal role in brain development. Indeed, recent increasing evidence has revealed diverse microglial functions at the embryonic stage. For example, microglia control differentiation of neural stem cells, regulate the population size of neural progenitors and modulate the positioning and function of neurons. Moreover, microglia exert functions not only on neural lineage cells but also on blood vessels, such as supporting vascular formation and integrity. This review summarizes recent advances in the understanding of microglial cellular dynamics and multifaceted functions in the developing brain, with particular focus on the embryonic stage, and discusses the fundamental molecular mechanisms underlying their behavior.

## 1. Introduction

Microglia are the resident immune cells in the central nervous system (CNS), comprising the brain, spinal cord, and retina. Microglia were initially identified as innate immune cells acting at the front line to defend against damage-related agents, and bacterial and viral invasion within the CNS (Ajami et al., 2018; Thorsdottir et al., 2019; Picard et al., 2021; Waltl and Kalinke, 2022). Microglia were once mainly assumed to function in pathological states, such as aging, chronic stress, and Alzheimer’s disease, by mediating neuroinflammatory processes (Tynan et al., 2010; Venegas et al., 2017; Streit et al., 2020). However, an increasing body of research has demonstrated their essential roles under physiological conditions: microglia support neuronal differentiation and synaptic organization, and maintain the CNS environment by removing cellular debris and apoptotic cells (Wake et al., 2009; Paolicelli et al., 2011; Parkhurst et al., 2013; Sierra et al., 2014; Shemer et al., 2015).

Although diverse microglial roles in the adult brain have been revealed, there has been relatively little research looking into microglia in the embryonic brain. Nonetheless, emerging evidence over the last 10 years has revealed that embryonic microglia, despite their scarce abundance (0.5–1.0% of total brain cells) (Hattori and Miyata, 2018), play an essential role in the developing brain as reviewed later. These cells interact with surrounding cells, i.e., neural lineage cells (neural progenitors and mature neurons) and vascular endothelial cells, in the developing cerebral wall through their extensive migration within the structure (Swinnen et al., 2013; Hattori et al., 2020). Such broad microglial patrolling activity enables them to exert various functions during neurogenesis and vascular formation in development.

Recently, multiple human studies reported that elevated maternal inflammation during pregnancy is associated with the emergence of separate psychological outcomes in their offspring later in development (Han et al., 2021; Kwon et al., 2022). Emerging evidence using direct assessment of inflammatory activation have linked maternal exposure to infection during pregnancy with greater likelihood of offspring psychiatric diagnoses of autism, schizophrenia, bipolar disorder, and major depressive disorder (Mac Giollabhui et al., 2019; Aguilar-Valles et al., 2020).

Of note, maternal inflammation has detrimental effects on fetal microglia. In mice, intraperitoneal challenge with lipopolysaccharide (LPS) during late gestation stages [at embryonic day (E) 15–17] induced fetal microglia to their proinflammatory state (Pont-Lezica et al., 2014; Schaafsma et al., 2017). Pont-Lezica et al. (2014) demonstrated that microglial dysfunction affected the formation of the corpus callosum, the largest commissure of the mammalian brain, using knockout mice of DAP12, a microglia-specific signaling molecule, and LPS-induced maternal inflammation mouse model. Ozaki et al. (2020) reported that maternal inflammation induced by intraperitoneal injection of poly(I:C) at E12 caused the sustained alterations in the patterns of microglial process motility and behavioral deficits in mice. Using maternal inflammation mouse models by LPS treatment, Hayes et al. (2022) demonstrated that gene expression alterations in microglia from neonatal whole brains compared to control animals treated with saline. Block et al. (2022) showed that gestational exposure to environmental toxins and socioeconomic showed male offspring displayed long-lasting behavioral abnormalities and alterations in the activity of brain networks encoding social interactions via an impairment of microglial synapse pruning.

These studies suggest that microglia are vulnerable during gestation, and maternal immune status is closely linked with the subsequent risks for cognitive disease in their offspring. Of note, microglia change their characteristics in a stage-dependent manner. Microglia present an amoeboid morphology, which is specific to immature microglia in the pallium in the early embryonic stage (Marin-Teva et al., 1998; Monier et al., 2007; Swinnen et al., 2013). Such morphology facilitates microglial flexible and locomotive migration (Swinnen et al., 2013). As development progresses, microglia transform into ramified shapes with long processes (Swinnen et al., 2013). Microglia also change their gene expression pattern over the course of development (Matcovitch-Natan et al., 2016; Thion et al., 2018; Hammond et al., 2019; Kracht et al., 2020). Thus, it is essential to carefully observe and investigate how fetal microglia act and function at each developmental stage in the physiological conditions to identify their abnormal behavior in the pathological states. This review focuses on the important studies on embryonic microglia and discusses the elusive points that remain to be elucidated in the future.

## 2. Microglial ontology and colonization into the CNS

**FIGURE 1:**
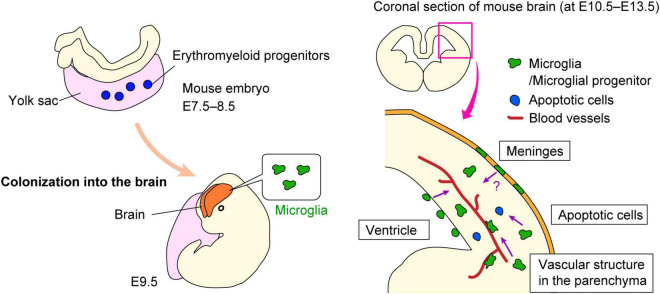
Microglial colonization into the brain. Schematic shows how microglial progenitors colonize the brain in mice. Microglial progenitors are generated in the yolk sac at E7.5–8.5, and then immigrate into the brain at E9.5. The possible mechanisms by which microglial precursors enter the brain remain are summarized.

Neural lineage cells including other glial cells (astrocytes and oligodendrocytes) originate from the neuroectoderm, whereas microglia are derived from erythromyeloid progenitors (EMPs) in the extraembryonic yolk sac blood island (Alliot et al., 1999; Ginhoux et al., 2010; Menassa and Gomez-Nicola, 2018). In mice, EMPs are generated at E7.5–8.5, and subsequently microglial precursors immigrate into the brain at E9.5 (Kierdorf et al., 2013; Prinz et al., 2021; Figure 1).

Although the mechanisms underlying the entrance of microglial precursors into the brain remain largely unknown, several possibilities have been suggested as far. First, blood vessels are considered one of the most likely candidates to support microglial colonization into the brain and retina. Early studies suggested that microglial appearance in the CNS coincides with the formation of vascular structure, as based on immunohistochemical analyses observing that microglia are distributed in the cerebral wall along with blood vessels (Ashwell, 1991; Cuadros et al., 1993; Rigato et al., 2011). Importantly, another study of microglial colonization demonstrated that *Ncx-1*-deficient mice that lack heartbeat and blood circulation because of a defect in sodium calcium exchanger 1, showed abnormally less microglial colonization of the brain from E9.5 to E10.5, whereas *Na^+^/Ca2^+^ exchanger-1* (*Ncx-1)*^+/+^ mice already had a substantial number of microglia at this stage (Ginhoux et al., 2010). They suggested that development of functional blood vessels was required for the recruitment of microglial progenitors from the yolk sac into the brain. In addition, a recent study showed that microglia start to colonize the retina along with blood vessel formation (Ranawat and Masai, 2021). They demonstrated that microglial precursors associated with hyaloid blood vessels initialize their infiltration into the retina by passing through the neurogenic regions in an IL-34/colony-stimulating factor-1 receptor (CSF1R)-dependent manner.

Second, the meninges may assist microglial infiltration into the cerebral wall. Several studies confirmed that microglial precursors accumulate in the meninges in the early embryonic stage, prior to their colonization of the cerebral wall (Boya et al., 1991; Navascues et al., 2000). Such regional information raised the possibility that microglia positioned in the meninges might infiltrate the brain parenchyma. A very recently published paper demonstrated that brain-border lymphatic vessels localize in the meninges support the early colonization of some microglial precursors into the embryonic zebrafish brain (Green et al., 2022). They discovered that Mrc1a^+^ microglial progenitors seed the brain parenchyma via lymphatic vasculature surrounding the brain before the entrance of traditionally described *Pu.1*^+^ microglia.

Third, pallial microglia might be provided from the ventricle. Previous study reported that macrophage progenitors first appeared in the fourth ventricle in chick embryos (Cuadros et al., 1993). These cells progressively appeared in the other brain region and in the spinal cord in the later stage. Earlier than this report, Kawaguchi (1980) proposed the model that ventricular microglia/macrophage precursors are derived from leptomeninges and arrived at the ventricle via the roof plate, based on their electron microscopic observation for amoeboid cells that were present in these regions (leptomeninges, ventricle, and roof plate) in chick embryos. In addition, Ashwell (1991) suggested a similar route of entry to explain the presence of microglia in rat forebrain. A recent study showed that the developing choroid plexus permits the entry of macrophages into the ventricle by secreting inflammatory molecules into the cerebrospinal fluid during maternal inflammatory states (Cui et al., 2020). Furthermore, our recent work based on brain slice culture and *in vivo* live imaging system indicated that intraventricular Mrc1 (CD206)^+^ macrophages, which are supplied from the center of the roof plate, infiltrate the developing cerebral wall at E12.5 in mouse brain (Hattori et al., 2023). Notably, the post-infiltrative cells subsequently acquired microglial properties [CD206^–^P2RY12 (purinergic receptor P2Y12)^+^], through GFP-labeled macrophage transplantation experiment and the tracking analysis for the intraventricular macrophages which were labeled with carboxyfluorescein succinimidyl ester (CFSE) (Hattori et al., 2023). In sum, some microglia in the pallium seem to be derived from intraventricular macrophages.

Fourth, apoptotic cells may recruit microglial progenitors. The colonization of the optic tectum by microglial progenitors is driven by apoptotic neuronal death, which occurs in the midbrain in the developmental process. The authors reported that lysophosphatidylcholine, a phospholipid released from apoptotic cells, promoted the entry of microglial precursors into the brain via its cognate receptors grp132b, indicating that microglial colonization of developing zebrafish midbrain is triggered by apoptotic neuronal death (Xu et al., 2016). The same group also reported that microglial precursors are attracted to the proximal brain regions via IL-34/CSF1R signaling, which is prior to neuronal apoptosis. In both *Il34*- and *Csf1ra*-deficient zebrafish larva, embryonic macrophages fail to migrate to the anterior head and colonize the CNS, showing that cytokine signaling support the colonization of microglia in collaboration with neuronal apoptosis in early zebrafish development (Wu et al., 2018).

Overall, several factors are involved in the colonization and entry of microglia/macrophage precursors into the developing CNS in a complicated manner. Further studies are needed to elucidate the cellular dynamics and mechanisms that regulate microglial colonization into the brain as well as the routes used by these cells.

## 3. Microglial distribution and migration in the brain parenchyma

Microglia account for about 10% of the total cells in the adult brain (Mittelbronn et al., 2001) but only 0.5–1.0% of cells in the embryonic brain (Hattori et al., 2020). Once microglia are seeded in the embryonic brain at E9.5, they increase the cell population through proliferation until the postnatal stage, which is induced by CSF-1, granulocyte macrophage colony-stimulating factor (G-CSF), neurotrophin-3, IL-4, and IL-5 (Navascues et al., 2000). The cell number of microglia reaches a peak in 2 weeks after birth in rodents, and microglial cell density is maintained with their low proliferation capacity until adulthood (Dalmau et al., 2003; Ginhoux et al., 2010; Arnoux et al., 2013; Nikodemova et al., 2015).

**FIGURE 2:**
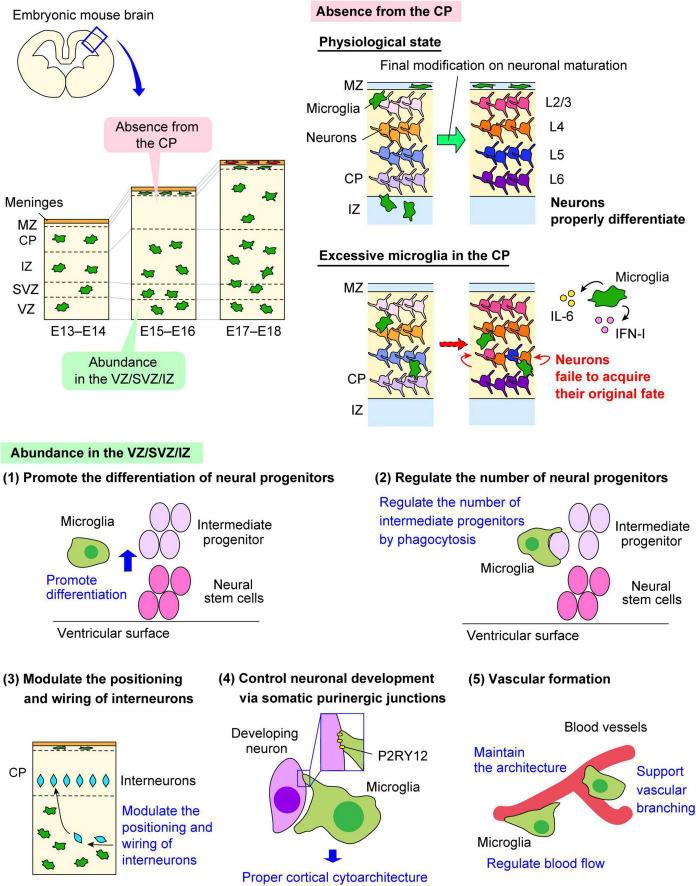
The multiple roles of embryonic microglia. The multifaceted functions of microglia in the embryonic brain are shown. Microglia change their distribution in a stage-dependent manner in the developing mouse cerebral wall. Microglia are homogenously distributed in the pallium in the early and late embryonic stages, but these cells are transiently absent from the CP from E15 to E16. Both microglial abundance in the VZ/SVZ/IZ and their transient absence from the CP are essential for the development of neural lineage cells and vascular formation. ATP, adenosine triphosphate; CP, cortical plate; CX3CL1, C-X3-C motif ligand 1; CX3CR1, C-X3-C motif receptor 1; IFN-I, type I interferon; IL-6, interleukin-6; IZ, intermediate zone; MZ, marginal zone; P2RY12, purinergic receptor P2Y12; SVZ, subventricular zone; VZ, ventricular zone.

In the developing brain, the distribution of microglia in the cerebral wall changes in a stage-dependent manner. Microglia are homogenously localized in the cerebral wall in the early embryonic stage. However, microglia transiently disappear from the cortical plate (CP), the region where mature post-migratory neurons accumulate after their radial migration, from E15 to E16. Instead, microglia preferentially colonize the ventricular zone (VZ), subventricular zone (SVZ), and intermediate zone (IZ). Intriguingly, microglia reenter the CP at E17 and then show a homogenous pattern toward the birth and postnatal stages (Cunningham et al., 2013; Swinnen et al., 2013; Hattori et al., 2020; Figure 2).

Although the molecular mechanisms that regulate microglial time-dependent migration remain unclear, emerging evidence is gradually unraveling them. Previous studies have suggested that microglial surrounding structure in the cerebral wall and chemokine molecules regulate their migration pattern.

First, the neural intermediate progenitors and the meninges regulate microglial stage-specific migration in the cerebral wall. During the embryogenesis, microglia most actively and extensively migrate throughout the cerebral wall at E14 (Swinnen et al., 2013; Hattori and Miyata, 2018). Our group previously reported that microglia show an interesting behavior at E14. Microglia do not randomly but in a regulatory manner migrate throughout the cerebral wall: microglia positioned in the CP migrate toward the meninges and then accumulate in the marginal zone, whereas the cells in the IZ move toward the SVZ. In the embryonic brain, C-X-C motif ligand 12 (CXCL12) is highly expressed in the cells in the meninges from E13, and the neural intermediate progenitors positioned in the SVZ at the strongest level at E14. On the other hand, microglia express C-X-C motif receptor 4 (CXCR4), the receptor for CXCL12 (Hattori et al., 2020). We found that microglia bidirectionally migrate in a CXCL12/CXCR4-dependent manner at E14, thereby permitting absence of microglia from the CP at E15.

Second, radial glial cells contribute to microglial migration. The fibers of radial glial cells regulate microglial migration in the quail retina (Sanchez-Lopez et al., 2004) and mouse embryonic spinal cord (Rigato et al., 2011). Sanchez-Lopez et al. (2004) reported that microglia spread in the tissue through their radial migration in the quail retina. Microglia use radial processes of s-laminin-expressing Müller glia as their foothold for their radial migration. Rigato et al. (2011) observed that microglia physically interact with radial glial cell fibers in the ventral area of the spinal cord in the embryonic mouse. Microglia start to colonize the mouse embryonic spinal cord at E11.5. At E13.5, microglia invade the ventral marginal zone along with radial glial cell fibers, which elongate toward the pial surface. Another study based on live imaging demonstrated that fetal microglia survey their environment in association with radial glia projections when an exogenous insult is induced (Rosin et al., 2021). Upon an insult [i.e., *in utero* electroporation or adeno-associated virus (AAV) transduction], hypothalamic microglia actively migrate by touching with radial glial cells that line the third ventricle of the E15.5 hypothalamus.

Third, the microglia-fibronectin interaction regulates microglial migration in a stage-dependent manner in the embryonic mouse brain (Smolders et al., 2017). At E13.5, α5β1 integrin, which is the receptor for the adhesion molecule fibronectin, facilitates microglial active migration. In contrast, microglia decrease their migration speed at E15.5 and E17.5 due to the decrease of cortical fibronectin production and α5β1 integrin expression on microglia.

Forth, blood vessels might be utilized as the scaffold of microglia to migrate throughout the CNS structure. In the postnatal and adult brain, microglia have been suggested to migrate along with blood vessels (Checchin et al., 2006; Fantin et al., 2010), it still remains unclear in the embryonic brain, though. But, about half of microglia are associated with blood vessels at E14 mouse brain (Hattori et al., 2022), raising the possibility that microglia may migrate along with blood vessels. In the spinal cord, microglia have been suggested to reach the spinal cord periphery through developing blood vessels in the embryonic mouse (Rigato et al., 2011).

Taken together, microglial distribution and migration in the developing CNS are spatiotemporally regulated by multiple mechanisms.

## 4. Microglial various effects on neural lineage cells

Active microglial motility enables these cells to associate with surrounding cells, such as neural lineage cells and vascular composing cells (Swinnen et al., 2013; Smolders et al., 2017; Hattori et al., 2020), and exert a pivotal role on them in the process of brain development (Figure 2).

### 4.1. Regulation of the differentiation and population size of neural progenitors

In the embryonic stage, microglia tend to colonize the VZ/SVZ, which is the neurogenic region of the brain in rodents and primates (Antony et al., 2011; Cunningham et al., 2013; Arno et al., 2014; Squarzoni et al., 2014). The knockout of CSF1R do not only target microglia because the CSF-1/CSF1R signaling is essential for the development of monocytes and other tissue-specific macrophages. However, the studies using CSF1R knockout animals raised the possibility that microglia influence neurogenesis in the embryonic brain.

Colony-stimulating factor-1 receptor deficient mice showed the abnormal brain architecture characterized with the reduced brain size, olfactory bulb atrophy and expansion of lateral ventricle size (Erblich et al., 2011; Nandi et al., 2012). In CSF1R knockdown zebrafish, retinal progenitor cells maintained their continuous state of proliferation, which led to the delayed onset of neurogenesis, indicating that microglia contribute to neurogenesis by inducing neural progenitors to exit the cell cycle (Huang et al., 2012). Of note, reoccupation of microglia in the retina partially recovered neurogenesis. Another study using cytomegalovirus (CMV) promoter-driven *Csf1r^flox/flox^* mice, in which microglia are conditionally depleted, the number of Tbr2^+^ intermediate progenitors positioned in the SVZ decreased in the mid and late embryonic stages (Arno et al., 2014). Supporting this finding, pharmacological depletion of microglia by the intraventricular administration of clodronate liposomes reduced the number of Tbr2^+^ neural intermediate progenitors in the SVZ and increased that of Pax6^+^ neural stem cells in the VZ in the mid embryonic stage in mice (Hattori and Miyata, 2018). Arrest of microglial motility by administration of the CXCR4 antagonist (AMD3100) resulted in a decrease of the number of Tbr2^+^ cells and an increase of Pax6^+^ cells. Furthermore, *in vitro* cell culture experiments demonstrated that the proportion of Tbr2^+^ cells in neural progenitors was increased when co-cultured with isolated microglia compared with cultures without microglia, whereas that of Pax6^+^ cells was decreased. The data indicate that microglia promote differentiation of neural stem cells into intermediate progenitors.

Microglia also regulate the cell population size of neural progenitors by phagocytosis. Cunningham et al. (2013) reported that microglia engulf neural progenitors to reduce their number in the developing rat brain. They showed that induction of a proinflammatory state in microglia through maternal inflammation using LPS led to a decrease in both Pax6^+^ and Tbr2^+^ cells in the VZ/SVZ, resulting in reduced thickness of the VZ/SVZ during the late embryonic period. In contrast, microglial inactivation by minocycline administration, induction to the anti-inflammatory state by doxycycline administration, and microglial depletion by liposomal clodronate increased the number of Pax6^+^ and Tbr2^+^ cells in the VZ/SVZ. They also demonstrated that microglia do not only engulf apoptotic cells but also live neural progenitors and mature neurons, suggesting that microglia do not merely eliminate the dead cells but actively modulate the cell population size. In line with this, another study also found that microglia with phagocytic features in the VZ/SVZ form a close connection with Tbr2^+^ neural progenitors, and their phagocytic activity was augmented in the inflammation model induced by the peritoneal administration of LPS to the mother mouse, leading to the decrease of Tbr2^+^ intermediate progenitors (Tronnes et al., 2016). A pre-exposure to progesterone, which reduces microglia activation, significantly increased the density of Tbr2^+^ cells compared to LPS treatment alone, which was similar to the density observed in control animals.

Taken together, microglia play an important role in regulating the size of the neural precursor pool by promoting the differentiation of neural progenitors and/or maintaining their total number in the developing brain.

### 4.2. Neuronal circuit formation

Most excitatory projection neurons are generated from neural stem cells positioned at the apical side of the neocortical primordium and migrate radially toward the basal surface (Rakic, 1974; Nadarajah et al., 2001), whereas inhibitory interneurons are produced in the ganglionic eminence and migrate tangentially toward the neocortex (Marin et al., 2001; Tamamaki et al., 2003; Lim et al., 2018). Embryonic microglia not only have an effect on the population size of neural progenitors but also restrict the outgrowth of dopaminergic axons of interneurons into the forebrain. A recent study reported that microglia regulate the entrance and positioning of interneurons (Squarzoni et al., 2014). The authors demonstrated that normal activity of microglia limits the embryonic outgrowth of dopaminergic axons in the forebrain. Moreover, they showed that microglial depletion and maternal immune activation remarkably affect the positioning of Lhx6^+^ interneurons, which are locally restricted in layer V of the neocortex. This interneuron subpopulation prematurely entered the neocortex, with a reduced focal distribution around layer V under these conditions. Furthermore, the authors demonstrated that microglia fine-tune neocortical interneuron positioning in a manner dependent on CX3CR1 and DNAX-activating protein of 12 kDa (DAP12) signaling.

As mentioned above, embryonic microglia change their distribution in a stage-dependent manner. Hattori et al. (2020) demonstrated that microglia bidirectionally migrate within the mid embryonic cerebral wall to permit temporal microglial absence from the CP at E15–E16 in the mouse embryonic brain. Through experiments to artificially expose post-migratory neurons positioned in the CP to excessive microglia *in vivo* or *in vitro* co-cultures of neurons with microglia, the authors showed that post-migratory neurons failed to appropriately express subtype-associated transcription factors; the cells displayed a tendency toward reduced expression of deep-layer neuron marker genes and increased expression of typical upper-layer neuron marker genes. Furthermore, they demonstrated that microglia-derived IL-6 and type I interferon (IFN-I) participate in the disturbance of gene expression of neuronal subtype-associated genes in post-migratory neurons. Hence, this transient microglial absence from E15 to E16 is required for post-migratory neurons in the CP to fine-tune expression of transcription factors needed for proper differentiation.

A recent study demonstrated that microglial processes form specialized contacts with the cell bodies of developing neurons throughout embryonic, early postnatal, and adult neurogenesis (Cserep et al., 2022). Such early developmental formation of microglia-neuron contact is associated with the somatic purinergic junctions which have a specialized nanoarchitecture optimized for purinergic signaling in the adult brain (Cserep et al., 2020). Furthermore, the formation and maintenance of these junctions is regulated by microglial P2RY12. Deletion of P2RY12 inhibited the proliferation of neuronal progenitors, which caused abnormal cortical structure in the developing and adult brain.

Together, how microglia interact with immature neurons in the developing brain and which type of cellular communication has an impact on neuronal complicated networks in the adult brain have been enthusiastically studied. Future studies to investigate the exact cellular communication pathways enabling microglia to influence the development of neurons and the formation of neural networks are required.

### 4.3. Gliogenesis

Recent studies have suggested a potential role of microglia in gliogenesis. An *in vitro* study showed that microglia-derived factors such as interleukin-6 (IL-6) and leukemia inhibitory factor (LIF) promoted the differentiation of *in vitro*-prepared neural stem/progenitor cells, which were obtained from the SVZ of rats on E16, into astrocytes (Nakanishi et al., 2007).

Several studies that focused on postnatal stages showed that microglia contribute to gliogenesis. Shigemoto-Mogami et al. (2014) reported that microglia modulate the survival and maturation of oligodendrocytes via releasing of IL-1β, IL-6, tumor necrosis factor alpha (TNF-α), and platelet-derived growth factor (PDGF) at the early postnatal stage. Nemes-Baren et al. (2020) demonstrated that microglia engulf oligodendrocyte progenitors (OPCs) in the corpus callosum during early postnatal development before myelination. Sherafat et al. (2021) reported that microglial neuropilin 1 promotes the expansion of OPCs in the white matter tracts during development and remyelination.

Although the studies which investigate microglial contribution to gliogenesis in the embryonic stage are few, a recent study suggested that embryonic microglia have an effect on gliogenesis in the developing hypothalamus (Marsters et al., 2020). Marsters et al. (2020) demonstrated that embryonic microglia influence nearby Olig2^+^ neural precursors through cytokine signaling and are required for proper maturation of oligodendrocytes in the developing tuberal hypothalamus. By E11.5, microglia invade the tuberal hypothalamus, and by E15.5 these cells accumulate alongside Olig2^+^ progenitors which are positioned in the hypothalamic sulcus, adjacent to the third ventricle. In microglial depletion model using a PLX5622, the authors observed a migration delay of Olig2^+^ cells from the VZ. It also caused a disruption of maturation and migration of OPCs in the gray matter at the embryonic and early postnatal stage. In particular, the authors found that C-C motif chemokine ligand 2 (CCL2) and CXCL10 derived from microglia influenced neuronal differentiation at the expense of astrocyte differentiation, with CCL2 further promoting an oligodendrocyte fate. Studies have shown that microglia in the forebrain SVZ take on a similar activated form during neurogenesis and gliogenesis. Thus, microglia may not only regulate the differentiation and function of neurons but also contribute to the fate decision of glial progenitors in the embryonic brain.

## 5. Microglial functions on blood vessels

Blood vessels play essential roles in microglial colonization and migration; microglia support blood vessel formation, and vice versa.

Previous study proposed that proper retinal blood vessel formation requires an adequate resident microglial population. Ischemic retinopathy mouse models exhibit decreased microglia concomitant with the characteristic reductions in vasculature observed in these retinopathies in the postnatal stage. Microglia depletion using liposomal clodronate impairs vascular development in the rat retina, suggesting that an adequate resident microglial population is critical for proper retinal blood vessel formation (Checchin et al., 2006). They also confirmed that microglia occupied the entire retinal surface in the human fetal retina at the 15-week of gestation, when retinal blood vessels are just beginning to emanate from the optic disc.

Subsequently, two important studies based on analysis of mutant mice with a deficiency in macrophage and microglial development showed that microglia contribute to vascular development. Kubota et al. (2009) found that CSF1-deficient mice resulted in defects in vascular and lymphatic development at the early postnatal stage. They demonstrated a significant decrease in branching of the primary vascular plexus in these mice. Fantin et al. (2010) found embryonic tissue macrophages in close spatiotemporal association with sprouting vessels in the embryonic mouse hindbrain. Microglia form the simultaneous connections with one or more spatially well-separated tip cells, and these cells promote fusion of vascular tip cells, which extend thin filopodia. Microglia numbers were correlated with numbers of vascular branch points, and were frequently found to be in contact with neighboring endothelial sprouts. They also observed that macrophages were also associated with endothelial tip cells during vessel fusion in the zebrafish trunk. Using CSF-1^op/op^ mice with an inactivating point mutation of CSF-1 and PU.1-deficient mice, they demonstrated that CSF-1 is an essential recruitment factor for embryonic macrophages in the mouse brain, which is consistent with the observations for zebrafish panther mutants, which lack a functional CSF1R. In CSF1R-deficient zebrafish, tissue macrophages could differentiate and migrate into the head mesenchyme, but their colonization into the brain was impaired.

An increasing body of studies based on the postnatal and adult brain have demonstrated that microglia contribute to vascular formation, such as extension and branching, vascular diameter modulation, regulation of blood flow circulation and blood brain barrier (BBB) integrity, but little research has focused on microglial function in the embryonic brain (Hattori, 2023). It is certainly worth investigating microglial function in such earlier stage to understand how abnormally activated microglia in the fetal brain affect vascular structure and function.

## 6. Conclusion

Microglial colonization of the brain parenchyma and their migration activity are tightly regulated in a spatiotemporal manner by a vast series of cues elicited from other surrounding CNS cells. Given that microglia contribute to a variety of steps in neurogenesis and vascular development, unnecessary microglial activation or disruption may cause microglial functional failure at critical time points during development, which is likely to lead to the aberrant brain formation and function. Loss of a functional Trem2 signaling pathway has been demonstrated as the genetic cause of Nasu-Hakola disease, which is characterized by early onset cognitive dementia (Thrash et al., 2009), suggesting that microglial proper functioning during development is crucial for the process of brain development. Moreover, recent studies have reported that maternal environmental factors, such as viral or bacterial infection, abnormal immune activation, or nutrition condition, are associated with fetal aberrant brain development and neurological disease manifestation in their offspring (Brown and Derkits, 2010; Kwon et al., 2022). Wu et al. (2017) demonstrated that lack of IL-6 signaling in placental trophoblasts effectively blocks inflammatory responses induced by maternal immune activation in the placenta and the fetal brain as well as downstream neuropathological and behavioral impairments using *Cyp19-Cre*: *Il6ra^fl/fl^* mice. Notably, embryonic microglia have been suggested to directly sense maternal inflammation and change their phenotype and/or motility in the embryonic stage (Schaafsma et al., 2017; Ozaki et al., 2020). When pregnant mice were treated with poly(I:C) at E12.5, fetal brain microglia isolated by the CD11b expression level obtained from E16 mice showed a significant increase of IL-6 production compared to those obtained from fetal brains in mock-treated pregnant mice (Pratt et al., 2013). To prevent the occurrence of neurological disorders in the offspring by maternal inflammation, further investigation for how maternal immune activation and the environmental factors affect the fetal microglia is needed. As mentioned above, microglia dramatically change their characteristics and behavior in a stage-dependent manner in the embryonic stage (Swinnen et al., 2013; Matcovitch-Natan et al., 2016; Hammond et al., 2019). Thus, it is quite important to carefully investigate how fetal microglia act and function at each developmental stage in the physiological conditions. The understanding for them in turn may contribute to identify microglial abnormal behavior of in the pathological context.

Furthermore, recent advances in single-cell transcriptomic analysis revealed that microglial have heterogenic properties in the spatial and developmental axes, and not only in the postnatal and adult brain but also in the embryonic brain (Matcovitch-Natan et al., 2016; Thion et al., 2018; Hammond et al., 2019; Kracht et al., 2020). We recently reported that microglia constitute the cells which use different colonization routes from the yolk sac into the embryonic cerebral wall (Hattori et al., 2023). To address how microglial heterogeneity is generated, we need to evaluate the possibility that differences in microglial colonization routes or entry timing into the pallium are related to their heterogeneity. Moreover, it is also interesting to investigate how maternal abnormal immune activation affects microglial colonization route and timing into the pallium. These investigations may provide profound insight into microglial developmental biology, and contribute to elucidation of the mechanism of brain development.

## Author contributions

YH wrote the manuscript and created the figures.
